# Triglyceride glucose-related indexes and lipid accumulation products—reliable markers of insulin resistance in the Chinese population

**DOI:** 10.3389/fnut.2024.1373039

**Published:** 2024-07-03

**Authors:** Lei Liu, Yufang Luo, Min Liu, Chenyi Tang, Hong Liu, Guo Feng, Meng Wang, Jinru Wu, Wei Zhang

**Affiliations:** ^1^Health Management Center, The Third Xiangya Hospital of Central South University, Changsha, China; ^2^Department of Clinical Nutrition, The Third Xiangya Hospital of Central South University, Changsha, China; ^3^Department of Clinical Nutrition, Hunan Aerospace Hospital, Changsha, China

**Keywords:** triglyceride glucose-related indexes, lipid accumulation products, insulin resistance, HOMA-IR, lipid-related indices

## Abstract

**Background:**

Insulin resistance (IR) is a pivotal pathogenic component of metabolic diseases. It is crucial to identify convenient and reliable indicators of insulin resistance for its early detection. This study aimed at assessing the predictive ability of seven novel obesity and lipid-related indices.

**Methods:**

A total of 5,847 female and 3,532 male healthy subjects were included in the study. The triglyceride glucose (TyG) index, TyG-body mass index (TyG-BMI), TyG-waist circumference (TyG-WC), lipid accumulation products (LAP), body roundness index (BRI), body adiposity index (BAI), and visceral adiposity index (VAI) were measured and calculated using the established formulae. IR was diagnosed using the homeostatic model assessment of insulin resistance (HOMA-IR) index over the third quantile.

**Results:**

The levels of all seven lipid-related indices were significantly higher in subjects with higher HOMA-IR values than in those with lower HOMA-IR values. These indices displayed moderate to high effectiveness [receiver operating characteristic (ROC) curve-area under the curve (AUC) > 0.6] in predicting IR. Among them, TyG-BMI (AUC: 0.729), LAP (AUC: 0.708), and TyG-WC (AUC: 0.698) showed the strongest association with HOMA-IR. In the female population, the AUC for TyG-BMI, LAP, and TyG-WC in predicting IR was 0.732, 0.705, and 0.718, respectively. Logistic regression analysis showed the optimal cut-off values of those indicators in predicting IR as follows: TyG-BMI: male subjects – 115.16 [odds ratio (OR) = 6.05, 95% CI: 5.09–7.19], female subjects – 101.58 (OR = 4.55, 95% CI: 4.00–5.16); LAP: male subjects – 25.99 (OR = 4.53, 95% CI: 3.82–5.38), female subjects – 16.11 (OR = 3.65, 95% CI: 3.22–4.14); and TyG-WC: male subjects – 409.43 (OR = 5.23, 95% CI: 4.48–6.24), female subjects – 342.48 (OR = 4.07, 95% CI: 3.59–4.61).

**Conclusion:**

TyG-index-related parameters and LAP appear to be effective predictors of IR in the Chinese population. Specifically, TyG-BMI may be the most appropriate predictor of IR.

## 1 Introduction

Insulin resistance (IR) is characterized by the inability of exogenous or endogenous insulin to perform its role in glucose uptake and utilization. It is the pivotal pathogenic factor for various conditions, such as type 2 diabetes mellitus (T2DM), metabolic syndrome, chronic kidney disease (1), and polycystic ovary syndrome. Furthermore, IR serves as the independent predictor of elevated cardiovascular risk (2–4). In these circumstances, higher insulin levels are needed to achieve glycemic control and prevent ketosis, thereby causing the compensatory secretion of excessive insulin to maintain blood glucose (5). Over-nourished, sedentary modern lifestyles significantly promote the incidence of metabolic diseases and subsequently increase medical expenses. According to the International Diabetes Federation, the global prevalence of diabetes was 8.8% (415 million) as of 2015 and is expected to increase to 10.4% (642 million) by 2040 (6). Currently, over 1 billion people across the globe suffer from metabolic syndrome (7). According to the latest research (8), the prevalence of metabolic syndrome among the population aged 20 years and above in China is as high as 31.1%. In addition, metabolic syndrome not only increases the incidence, morbidity, and mortality rates of T2DM but also imposes a heavy burden on patients, families, and society (9). Metabolic syndrome and related diseases increase the total cost of medical care by 60% (10). Given that a large population is affected by metabolic syndrome, along with the health hazards and economic expenses it brings, this issue cannot be ignored and must be given sufficient attention. Therefore, early diagnosis of IR warrants significant attention.

The gold standard for diagnosing IR is based on the hyperglycemic clamp assay, which requires intravenous glucose infusion and frequent monitoring of plasma glucose. Owing to the complexity and time-consuming nature of these procedures, numerous studies have focused on identifying simpler and effective alternatives for early diagnosis of patients suffering from IR. Among these alternatives, the most widely used indicator for IR is the homeostatic model assessment of insulin resistance (HOMA-IR), an index calculated using fasting insulin (FINS) and fasting plasma glucose (FPG). However, there are some limitations to its clinical use. For instance, there are no instruments for measuring serum insulin in community hospitals, especially in developing countries. In addition, the cost of serum insulin testing is relatively high. Therefore, it is of great importance to identify some predictive indicators that are both convenient and accurate.

Recently, some simple anthropometric and biochemical indicators, along with combinations of these indicators, have been developed to assess the IR risk. Obesity and lipid-related indices, including the visceral adiposity index (VAI) (11), triglyceride glucose (TyG) index (12), TyG-index-related parameters (TyG-body mass index [TyG-BMI]), TyG-waist circumference (TyG-WC)] (13, 14), body roundness index (BRI) (15), lipid accumulation products (LAP) (16), and body adiposity index (BAI) (15), are potential IR predictors and are widely discussed in epidemiological studies. Currently, there are only a few comparative studies focused on predicting IR through obesity and lipid-related indices. Additionally, the conclusions drawn from some of these existing studies are controversial. The purposes of this study are as follows: (1) to assess the performance and reliability of VAI, LAP, BAI, BRI, TyG, TyG-BMI, and TyG-WC as predictors of IR and (2) to evaluate the diagnostic accuracy of these indexes in identifying IR in the Chinese population.

## 2 Materials and methods

### 2.1 Study population

**Figure 1:**
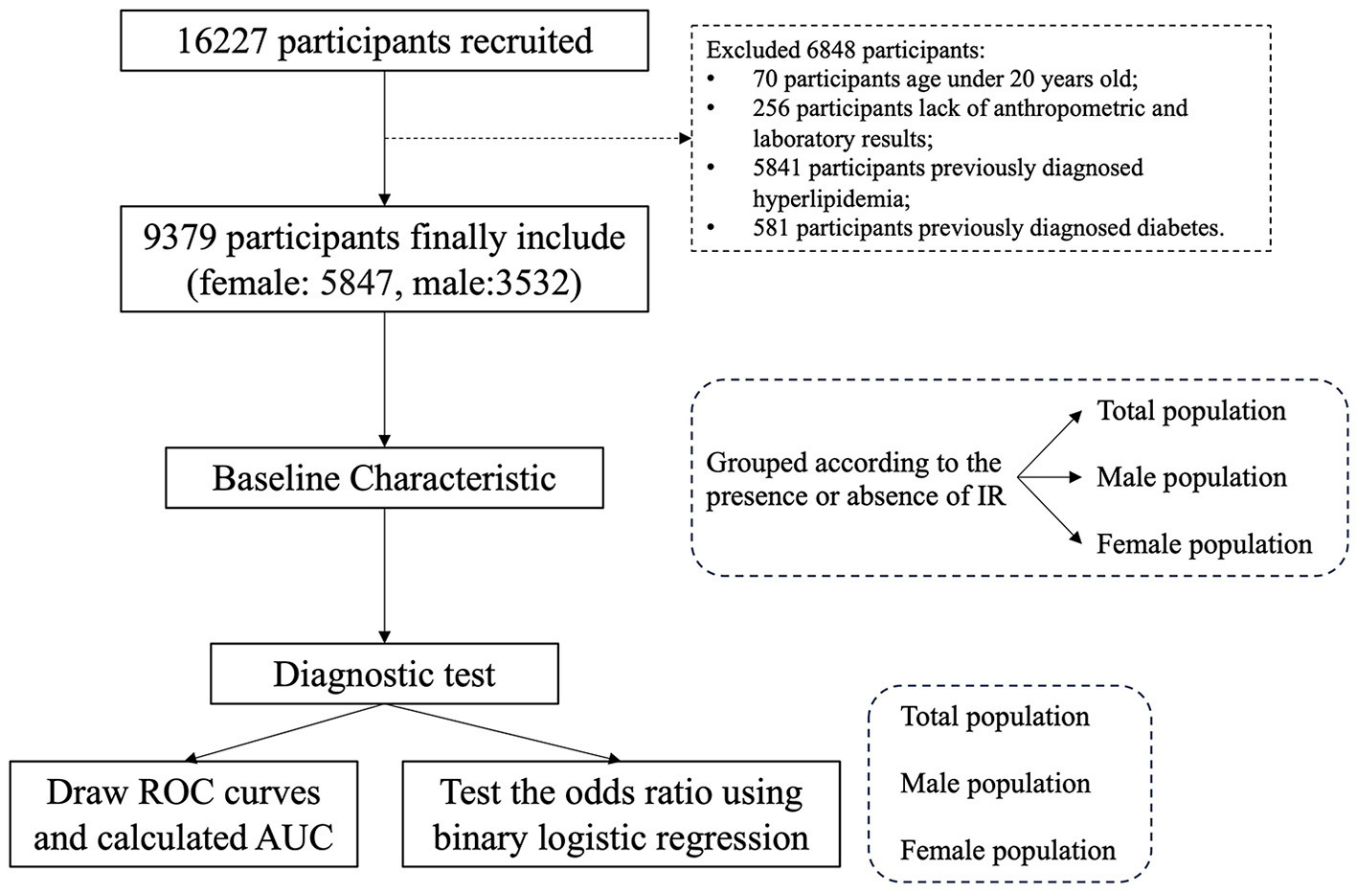
Flowcharts.

Data were collected from the Health Management Center of the Third Xiangya Hospital of Central South University from 2018 to 2021. There were 16,227 participants included in the primary database. Excluding 70 participants under 20 years of age, 256 participants without anthropometric results and fasting laboratory examination data, 5,841 participants with hyperlipidemia, and 581 participants with previous diagnoses of diabetes, 9,379 participants were ultimately included in the study (Figure 1). The research was conducted in accordance with the Declaration of Helsinki and was approved by the Institutional Review Board of the Third Xiangya Hospital of Central South University (Approval no. 22194). All procedures were carried out with the adequate understanding and written consent of the subjects.

### 2.2 Anthropometric and laboratory measurements

Body weight was measured using a weighing scale (SECA Model 803, Hamburg, Germany) to the nearest 0.1 kg, and height was measured using a stadiometer (SECA Model 213, Hamburg, Germany) to the nearest 0.1 cm. WC was measured to the nearest 0.1 cm, taken above the uppermost lateral border of the right ilium. For laboratory tests, the subjects fasted for 9–12 h before blood collection. Data on total cholesterol (TC), high-density lipoprotein cholesterol (HDL-c), low-density lipoprotein cholesterol (LDL-c), triglycerides (TG), FINS, and FPG were collected.

### 2.3 Calculation of parameters for insulin resistance

IR was defined using the HOMA-IR index over the third quantile. Parameters for IR are calculated as follows:

HOMA-IR = FINS (μIU/mL) × FPG (mmol/L)/22.5
VAI for men = [WC (cm)/(39.69 + 1.88 × BMI)] × TG (mmol/L)/1.03 × [1.31/HDL-c(mmol/L)]
VAI for women = [WC (cm)/(36.58 + 1.89 × BMI)] × TG (mmol/L)/0.81 × [1.51/HDL-c(mmol/L)]
BAI = hip circumference (HC)/Height^1.5^−18
BRI $=\sqrt{1-\left(\frac{WC-2\pi x^{2}}{0.5heghtx^{2}}\right)}$
LAP for men = [waist circumference (cm)−65] × [triglyceride concentration (mmol/L)]
LAP for women = [waist circumference (cm)-58] × [triglyceride concentration (mmol/L)]
TyG-index = Ln [fasting triglycerides (mg/dL) × fasting glucose (mg/dL)/2]
TyG-BMI = Ln [fasting triglycerides (mg/dL) × fasting glucose (mg/dL)/2] × BMI
TyG-WC = Ln [fasting triglycerides (mg/dL) × fasting glucose (mg/dL)/2] × WC (cm)

### 2.4 Statistical analysis

Data were analyzed using Statistical Package for the Social Sciences (SPSS), Statistics Version 26.0 (IBM Co., Armonk, NY, USA). The data were first tested for normality. For data with a normal distribution or approximately normal distribution, comparisons of counting data were conducted using the chi-squared test, and the grouped *t*-test was used for the comparison between the two groups. The receiver operating characteristic (ROC) curve is used to determine the area under the curve (AUC), sensitivity, and specificity of indirect prediction indicators for insulin resistance prediction. The cut-off value is selected as the value of the predictive indicator when the Jordan index (sensitivity + specificity −1) is at its maximum. Logistic regression analysis is used to analyze the odds ratio (OR) of insulin resistance when the indirect predictive indicators exceed cut-off values. *P* < 0.05 can be considered a statistically significant difference.

## 3 Results

### 3.1 Characteristics of participants

The present study ultimately included a total of 9,379 participants (5,847 female and 3,532 male participants), and the selection process is shown in Figure 1. Participants were grouped according to the presence or absence of insulin resistance (Table 1). The average age of the normal group is 42.2 ± 11.8 years, with 2,649 male participants (37.66%), while the average age of the IR group is 42.1 ± 11.6 years, with 883 male participants (37.65%). There was no significant difference in age and gender between the two groups. Compared with the normal group, participants in the IR group had significantly higher BMI, WC, HC, FPG, FINS, TG, LDL-c, HOMA-IR, BAI, BRI, LAP, VAI, TyG-index, TyG-BMI, and TyG-WC values, while HDL-c levels were significantly lower than those in the control group.

**Table 1:** Baseline characteristics of the participants.

**Characteristics**	**Normal (*n =* 7,034)**	**Insulin resistance (*n =* 2,345)**	***P*-value**
Age	42.2 ± 11.8	42.1 ± 11.6	0.200
Gender (men)	2,649 (37.66%)	883 (37.65%)	0.996
Gender (women)	4,385 (62.34%)	1,462 (62.35%)	0.996
BMI (kg/m^2^)	22.42 ± 2.75	24.84 ± 3.34	<0.001
WC (cm)	77.01 ± 8.9	83.46 ± 10.47	<0.001
HC (cm)	91.92 ± 5.24	95.86 ± 6.10	<0.001
FPG (mmol/L)	5.18 ± 0.46	5.54 ± 0.51	<0.001
FINS (uU/ml)	4.54 ± 1.46	10.72 ± 3.62	<0.001
TC (mmol/L)	4.77 ± 0.69	4.81 ± 0.80	0.037
TG (mmol/L)	1.06 ± 0.44	1.28 ± 0.47	<0.001
HDL-c (mmol/L)	1.45 ± 0.26	1.35 ± 0.22	<0.001
LDL-c (mmol/L)	2.76 ± 0.60	2.80 ± 0.63	0.015
HOMA-IR	1.05 ± 0.35	2.64 ± 0.97	<0.001
BAI	26.75 ± 3.08	28.36 ± 3.40	<0.001
BRI	2.97 ± 0.92	3.68 ± 1.15	<0.001
LAP	18.61 ± 13.62	30.87 ± 18.53	<0.001
VAI	1.18 ± 0.57	1.57 ± 0.68	<0.001
TyG-index	4.50 ± 0.22	4.63 ± 0.21	<0.001
TyG-BMI	101.01 ± 15.04	115.31 ± 18.06	<0.001
TyG-WC	347.09 ± 50.48	387.44 ± 57.60	<0.001

Data are presented as mean ± standard deviation or number (%). Insulin resistance was defined as a HOMA-IR value above the 75th percentile for each gender. BMI, body mass index; WC, waist circumference; HC, hip circumference; FPG, fasting plasma glucose; FINS, fasting insulin level; TC, cholesterol; TG, triglyceride; HDL-c, high-density lipoprotein cholesterol; LDL-c, low-density lipoprotein cholesterol; HOMA-IR, homeostatic model assessment of insulin resistance; LAP, lipid accumulation products; BRI, body roundness index; BAI, body adiposity index; VAI, visceral adiposity index; TyG-index, triglyceride-glucose index; TyG-BMI, TyG-body mass index; TyG-WC, TyG-waist circumference.

We also observed the distribution comparison of HOMA-IR, BMI, and the seven indirect predictive indicators of insulin resistance between different genders (Table 2).

**Table 2:** Distribution of indirect parameters for insulin resistance according to gender.

**Indexes**	**Men (*N =* 3,532)**	**Women (*N =* 5,847)**
HOMA-IR	1.43 ± 0.96	1.45 ± 0.86
BMI	24.36 ± 3.07	22.21 ± 2.81
BAI	25.56 ± 2.78	28.11 ± 3.11
BRI	3.55 ± 1.00	2.90 ± 0.97
LAP	27.87 ± 17.15	17.93 ± 13.83
VAI	1.31 ± 0.56	1.26 ± 0.66
TyG-index	4.62 ± 0.21	4.47 ± 0.22
TyG-BMI	112.84 ± 16.51	99.59 ± 15.28
TyG-WC	395.61 ± 49.38	333.96 ± 44.71i

Data are presented as mean ± standard deviation. HOMA-IR, homeostatic model assessment of insulin resistance; BMI, body mass index; BAI, body adiposity index; BRI, body roundness index; LAP, lipid accumulation products; VAI, visceral adiposity index; TyG-index, triglyceride-glucose index; TyG-BMI, TyG-body mass index; TyG-WC, TyG-waist circumference.

### 3.2 ROC-AUC of lipid predictors

**Figure 2:**
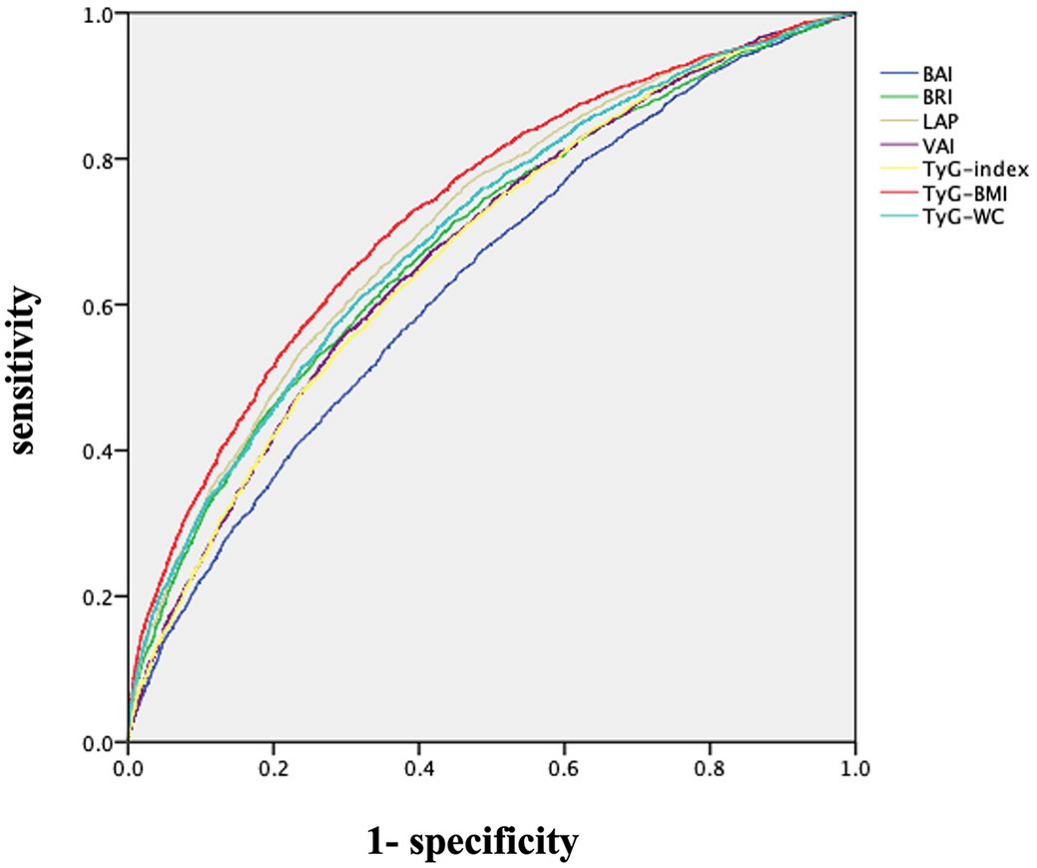
ROC curves of indirect parameters for insulin resistance.

ROC curves were drawn to determine the diagnostic value of the seven indirect indicators for insulin resistance (Figure 2). The results showed that all indicators displayed moderate to high values (ROC-AUC > 0.6). Among these indicators, TyG-BMI (AUC: 0.729), LAP (AUC: 0.708), and TyG-WC (AUC: 0.698) displayed the highest values in predicting insulin resistance (Table 3).

**Table 3:** The area under the curve for each parameter for insulin resistance according to gender.

	**Total (95% CI)**	**Men (95% CI)**	**Women (95% CI)**
BAI	0.635 (0.623–0.648)	0.673 (0.653–0.690)	0.636 (0.619–0.652)
BRI	0.685 (0.673–0.698)	0.733 (0.715–0.752)	0.674 (0.657–0.690)
LAP	0.708 (0.696–0.720)	0.742 (0.724–0.760)	0.705 (0.689–0.720)
VAI	0.674 (0.662–0.687)	0.668 (0.648–0.687)	0.678 (0.662–0.693)
TyG-index	0.672 (0.660–0.685)	0.672 (0.652–0.692)	0.687 (0.671–0.702)
TyG-BMI	0.729 (0.717–0.741)	0.780 (0.763–0.797)	0.732 (0.717–0.747)
TyG-WC	0.698 (0.686–0.710)	0.772 (0.755–0.790)	0.718 (0.702–0.733)

BAI, body adiposity index; BRI, body roundness index; LAP, lipid accumulation products; VAI, visceral adiposity index; TyG-index, triglyceride-glucose index; TyG-BMI, TyG-bodymass index; TyG-WC, TyG-waist circumference.

We investigated whether the results were applicable to both female and male groups. Subgroup analysis revealed that TyG-BMI, LAP, and TyG-WC had the highest AUC in both the male and female groups. The optimal cut-off values for these indicators in predicting IR are as follows (Table 4): TyG-BMI: male subjects 115.16, female subjects 101.58; LAP: male subjects 25.99, female subjects 16.11; and TyG-WC: male subjects 409.43, female subjects 342.48.

**Table 4:** Cut-off values for each parameter with their corresponding sensitivity, specificity, and odds ratio of insulin resistance according to gender.

**Indirect parameters for insulin resistance**	**Men**		**Women**	
	**Cut-off values (sensitivity and specificity)**	**Odds ratio (95% CI)**	**Cut-off values (sensitivity and specificity)**	**Odds ratio (95% CI)**
BAI	25.56 (69.4,57.5)	3.05 (2.59,3.59)	28.98 (50.0,70.2)	2.35 (2.08,2.65)
BRI	3.68 (69.4,65.6)	4.32 (3.67,5.10)	2.87 (64.0,62.4)	2.94 (2.60,3.33)
LAP	25.99 (74.9,60.4)	4.53 (3.82,5.38)	16.11 (66.5,64.8)	3.65 (3.22,4.14)
VAI	1.28 (69.0,57.7)	3.03 (2.57,3.56)	1.40 (52.7,73.7)	3.10 (2.74,3.51)
TyG-index	4.66 (67.2,59.5)	2.97 (2.53,3.48)	4.51 (62.3,65.0)	3.01 (2.66,3.41)
TyG-BMI	115.16 (75.3,66.5)	6.05 (5.09,7.19)	101.58 (66.3,69.8)	4.55 (4.00,5.16)
TyG-WC	409.43 (68.9,70.5)	5.23 (4.48,6.24)	342.48 (62.8,70.7)	4.07 (3.59,4.61)

OR, odds ratio; BAI, body adiposity index; BRI, body roundness index; LAP, lipid accumulation products; VAI, visceral adiposity index; TyG-index, triglyceride-glucose index; TyG-BMI, TyG-body mass index; TyG-WC, TyG-waist circumference.

### 3.3 Binary logistic regression analysis of lipid indicators

The OR of insulin resistance according to gender was tested using binary logistic regression and is shown in Table 4. TyG-BMI, TyG-WC, and LAP had the highest OR for IR, among which TyG-BMI had the highest dominance ratio both in men (OR = 6.05, 95% CI: 5.09–7.19) and in women (OR = 4.55, 95% CI: 4.00–5.16). TyG-WC had OR in male subjects (OR = 5.23, 95% CI: 4.48–6.24) and women (OR = 4.07, 95% CI: 3.59–4.61). LAP had OR in male subjects (OR = 4.53, 95% CI: 3.82–5.38) and women (OR = 3.65, 95% CI: 3.22–4.14).

## 4 Discussion

IR is the pivotal pathogenic component of many metabolic diseases, such as metabolic syndrome (5). The hyper-insulinemic euglycemic clamp, the gold standard diagnostic test for IR, is complex to perform, thereby limiting its application in clinical use. Although the HOMA-IR index is widely used, it also has shortcomings, including the potential for disturbance of exogenous insulin, unavailability and unstandardized laboratory measurement of plasma insulin, and high costs (17). Therefore, scientists have tried to explore new indicators of IR in recent decades, such as VAI, BRI, BAI, LAP, and TyG-related indexes (TyG-BMI and TyG-WC).

Amato et al. found that there is a significant negative correlation between VAI and insulin sensitivity, as measured by the hyperglycemic clamp assay (18). VAI has been proven to be a reliable positive indicator of diabetes (19), insulin-resistance-related diseases such as polycystic ovary syndrome (20), and cardiometabolic risk (18). Although it was first proposed for use in Caucasians, its effectiveness has also been verified in the Chinese population recently (19). In the present study, we found that individuals with IR have higher VAI values. Specifically, men with a VAI value exceeding 1.28 and women with a VAI value exceeding 1.40 have a three-fold increased risk of developing IR.

BAI and BRI are widely used in obesity assessment. In American cohorts, BAI performs better in assessing percent body fat than BMI. However, the accuracy of BAI in various ethnicities is not superior to BMI and WC (15). Similarly, a previous Chinese study revealed that BRI and BAI in predicting IR or DM are not superior to traditional indexes, including BMI and WC (15). This may be explained by the fact that the formula for these indices was developed based on the Western population, which has different body shapes compared with the East Asian population. In this study, we found that the diagnostic value of BAI and BRI for predicting IR is moderate.

A previous study revealed that LAP, calculated using WC and TG (21), is a reliable index of insulin resistance (22–24). The present study showed that LAP has a high predictive value for IR. Men with an LAP value exceeding 25.99 have a 4.53-fold increased risk of developing IR, and women with an LAP value exceeding 16.11 have a 3.65-fold increased risk of developing IR. Consistent with our results, Huang et al. found that LAP is a potential surrogate marker of IR compared with VAI in the Chinese population (25). However, compared with TyG-index-related parameters, especially TyG-BMI, LAP showed a relatively weak predictive value.

It has been reported that the accuracy of the TyG-index is consistent with the hyperglycemic clamp assay (26, 27), and the TyG-index is used to predict T2DM in the Asian population (28). Salazar et al. (29) have proposed a single cut-off point of 4.5 to classify Venezuelan individuals with IR. In the present study, the optimal cut-off values of the TyG-index for predicting IR were 4.66 (OR: 2.97, 95% CI: 2.53–3.48), with a sensitivity of 67.2% and a specificity of 59.5% in men, and 4.51 (OR: 3.01, 95% CI: 2.66–3.41), with a sensitivity of 62.3% and a specificity of 65.0% in women. Although the cut-off value of the TyG-index was close to that found in Salazar's research, the AUC was lower in the present study. We considered that it may cause differences between studies. Compared with Caucasians, Asians had a greater propensity to beta-cell dysfunction (30). In addition, ecological, environmental, lifestyle, and other factors may be attributed to the differences. Recently, TyG combined with BMI or WC has the best predictive value in IR diagnosis compared with other indices (24, 31). Sheng et al. (32) proposed that the TyG-index-related parameters have the high predictive value for predicting non-alcoholic fatty liver disease in the Chinese population. The present study verified that TyG-BMI has the highest predictive value compared with other indexes in large-scale Chinese populations. Men with a TyG-BMI value exceeding 115.16 have a 6.05-fold increased risk of developing IR, and women with a TyG-BMI value exceeding 101.58 have a 4.55-fold increased risk of developing IR, respectively.

Molecular mechanisms include the role of IR in vascular function, macrophage accumulation, and atherosclerosis progression (33). Among the IR indices, HOMA-IR only includes the plasma-related index and FINS level, while the calculation of LAP and TyG-index includes triglycerides. Apart from better beta-cell dysfunction in Asians compared with Caucasians (30), South Asians had more visceral adiposity and greater IR than East Asians, who tend to have a lower body mass index (BMI) and low insulin secretion (34). As a result, insulin levels may be significantly different among different ethnicities, which leads to different cut-off points of the HOMA-IR index in predicting IR (ranging from 1.22 to 2.48 with ROC methods on various populations) (2). On the other hand, triglycerides increase free fatty acids, resulting in increased transfer of free fatty acids from adipose to non-adipose tissues, which causes insulin resistance. Hypertriglyceridemia causes increased transport of free fatty acids to the liver, resulting in high hepatic glucose output. High levels of triglycerides in the liver and muscles can interfere with glucose metabolism in each target organ. Therefore, triglycerides play an important role in IR prediction. Furthermore, the TyG-BMI index includes BMI, which reflects total body fat, while the calculation of LAP and TyG-WC also includes waist circumference, which reflects visceral fat. We consider that including these measures will make the indicators more accurate in predicting IR. We have added this to the discussion section.

## 5 Conclusion

In conclusion, the present study suggests that TyG-BMI and LAP are ideal surrogate markers for predicting insulin resistance. In clinical practice, these indices could be integrated into routine metabolic assessments. The LAP and TyG-related indexes are more convenient for routine screening in clinical practice than the HOMA-IR index. The tests for triglyceride and fasting glucose and the measurement of anthropometry could be easily obtained even in primary medical institutions. Therefore, we suggest popularizing the calculation of LAP and TyG-related indexes in medical institutions, especially in patients with IR risk factors, to facilitate early diagnosis of IR.

The current research has several advantages. First, this study is based on large-scale data from the Chinese health population and includes subgroup analyses based on gender. In addition, this study proposes effective threshold values for each of the seven indices, which can serve as a reference for identifying populations at risk of IR in clinical applications. However, considering that this is a cross-sectional study, limitations such as the inability to establish causality exist. Therefore, further prospective studies are needed to verify the relationship between each alternative measure and cardiovascular risk factors.

## Data availability statement

The raw data supporting the conclusions of this article will be made available by the authors, without undue reservation.

## Ethics statement

The studies involving humans were approved by the Institutional Review Boards of the Third Xiangya Hospital of Central South University. The studies were conducted in accordance with the local legislation and institutional requirements. Written informed consent for participation in this study was provided by the participants' legal guardians/next of kin.

## Author contributions

LL: Data curation, Writing—original draft. YL: Writing—original draft, Validation. ML: Conceptualization, Writing—review & editing. CT: Writing—review & editing. HL: Formal analysis, Writing—original draft. GF: Formal analysis, Writing—original draft. MW: Funding acquisition, Writing—original draft. JW: Methodology, Writing—original draft. WZ: Conceptualization, Writing—original draft.
